# Social dynamics obscure the effect of temperature on air breathing in *Corydoras* catfish

**DOI:** 10.1242/jeb.222133

**Published:** 2020-11-12

**Authors:** Mar Pineda, Isabel Aragao, David J. McKenzie, Shaun S. Killen

**Affiliations:** 1 Institute of Biodiversity, Animal Health and Comparative Medicine, College of Medical, Veterinary and Life Sciences, University of Glasgow, Glasgow G12 8QQ, UK; 2 MARBEC, Université de Montpellier, CNRS, Ifremer, IRD, 9190 Montpellier, France

**Keywords:** Air-breathing fish, Environmental stress, Metabolic rate, Oxygen, Social behaviour

## Abstract

In some fishes, the ability to breathe air has evolved to overcome constraints in hypoxic environments but comes at a cost of increased predation. To reduce this risk, some species perform group air breathing. Temperature may also affect the frequency of air breathing in fishes, but this topic has received relatively little research attention. This study examined how acclimation temperature and acute exposure to hypoxia affected the air-breathing behaviour of a social catfish, the bronze corydoras *Corydoras aeneus*, and aimed to determine whether individual oxygen demand influenced the behaviour of entire groups. Groups of seven fish were observed in an arena to measure air-breathing frequency of individuals and consequent group air-breathing behaviour, under three oxygen concentrations (100%, 60% and 20% air saturation) and two acclimation temperatures (25 and 30°C). Intermittent flow respirometry was used to estimate oxygen demand of individuals. Increasingly severe hypoxia increased air breathing at the individual and group levels. Although there were minimal differences in air-breathing frequency among individuals in response to an increase in temperature, the effect of temperature that did exist manifested as an increase in group air-breathing frequency at 30°C. Groups that were more socially cohesive during routine activity took more breaths but, in most cases, air breathing among individuals was not temporally clustered. There was no association between an individual's oxygen demand and its air-breathing frequency in a group. For *C.* *aeneus*, although air-breathing frequency is influenced by hypoxia, behavioural variation among groups could explain the small overall effect of temperature on group air-breathing frequency.

## Abstract (precis)

**Summary:** Temperature effects on air breathing in an air-breathing fish are not consistent at the individual and group levels, suggesting that social dynamics can obscure links between physiology and behaviour.

## INTRODUCTION

Group-living is widespread throughout the animal kingdom and is associated with a variety of costs and benefits (Ward and Webster, 2016). Living in a group can be detrimental due to increased risk of competition (Rubenstein, 1978), parasitism (Rifkin et al., 2012) and disease (Krause and Ruxton, 2002). Despite this, individuals within groups can obtain significant benefits. Resource acquisition is often cited as a key benefit of group-living, but the focus has predominantly been on foraging (Killen et al., 2018; McKenzie et al., 2016). However, one resource that is vital for survival yet often overlooked, is oxygen. In some environments and for some taxa, sufficient oxygen can be especially difficult to obtain. For example, tropical freshwater environments can often become oxygen poor, or hypoxic. Although some animals have developed mechanisms to escape hypoxia (Graham, 1994) others, such as pond-dwelling fishes, are often unable to escape. It is in these hypoxic and closed environments where it is thought that the ability to breathe air in some fishes first evolved.

The morphological and physiological specialisations for air breathing are generally well established, yet little is known about the behavioural and ecological aspects of air breathing in fishes. To breathe air, an individual must surface, rendering them visible to aerial and terrestrial predators (Kramer and Graham, 1976). Consequently, air-breathing fishes can have disproportionately high predation rates compared with non-air-breathing species that live in the same habitat (Biro et al., 2004; Kramer and Braun, 1983; Mangel and Stamps, 2001). To offset the risk associated with surfacing, some air-breathing species engage in group air breathing. This social surfacing reduces the risk of predation for each individual (Kramer and Graham, 1976) and results in temporally clustered air-breathing events known as synchronised air breathing (Hill, 1972; Killen et al., 2018; Kramer and Graham, 1976; Shlaifer and Breder, 1940).

The occurrence of social air-breathing is remarkable given that groups consist of individuals with differing oxygen requirements (McKenzie et al., 2016). Within animal species, there is considerable among-individual variation in the minimal metabolic rate needed to sustain life (standard metabolic rate in ectotherms; SMR). In air-breathing fishes, individuals with a higher basal oxygen demand may be driven to breathe air more often than those with a lower oxygen demand (Lefevre et al., 2016; McKenzie et al., 2016). There is also wide variation among individuals in the proportion of baseline oxygen requirements that are met by air breathing. In *Clarias gariepinus*, for example, individuals vary between approximately 25 and 80% of the oxygen required for SMR coming from air (Mckenzie et al., 2016). Thus, when coordinating social air-breathing, individuals may compromise their own oxygen demand or willingness to perform risky air breathing to remain within a group (Killen et al., 2018). One of the major questions in air-breathing fish research, therefore, is to determine whether key individuals play a significant role in shaping the behaviour of entire groups. Knowing more about this mechanism could then, in turn, provide insight into the importance of individual heterogeneity in the overall functioning of social groups across taxa (Jolles et al., 2020). There has been a steady increase in research into individual variation and the impact of key individuals on overall group behaviour (Modlmeier et al., 2014), spanning a large variety of taxa (Ballard and Robel, 2012; Dornhaus et al., 2008; Knörnschild et al., 2010), with potentially important effects on a range of ecological phenomena including group movements and spatial positioning (Killen et al., 2012; Killen et al., 2017; McLean et al., 2018), sociability and dominance (Killen et al., 2016; Metcalfe et al., 1995), and group decision-making, foraging and learning (Mamuneas et al., 2015). Due to their acute need for oxygen, an easily quantifiable air-breathing response, air-breathing fishes provide an excellent model for studying the role of the individual in the functioning of social groups.

Central to the expression of physiological and behavioural traits, however, is the environment. For example, both oxygen availability and water temperature induce a wide range of physiological and behavioural responses in fishes (Clark et al., 2013; Domenici et al., 2007; Domenici et al., 2017; Killen et al., 2013). In animal social groups, acute hypoxia can decrease social cohesion and coordination among individuals (Domenici et al., 2017). For air-breathing fishes, any reduction in social group cohesion (specifically, an increase in distance between individual group members) could reduce the ability to perform coordinated group air-breathing. Oxygen availability in water is a clear driver of air breathing in bimodal fishes, given its role in the evolution of air breathing in the first place. However, the effect of hypoxic stress on air-breathing is often measured independently of temperature. This is surprising, given that both hypoxia and increased temperature can act together as a combined stressor (Graham et al., 1978), particularly in aquatic environments in response to climate change (Breitburg et al., 2018). The influence of temperature on air-breathing behaviour has been largely overlooked, despite it being a factor that increases oxygen demand. In one of the only studies to examine the effects of temperature on air-breathing in fishes, air-breathing frequency increased under a combination of elevated temperature and hypoxia, but only after prolonged exposure (Wang and Lin, 2018). Johansen et al. (1970) observed that air breathing increased in response to hypoxia and especially at warmer temperatures, although fish in this study were exposed to acute changes in temperature. Overall, little is known about the effects of temperature on air breathing and, in particular, whether impacts on the physiology of individuals in turn influence the behaviour of entire groups (Jolles et al., 2020).

We therefore studied the air-breathing behaviour of bronze corydoras catfish *Corydoras aeneus* at different oxygen availabilities and acclimation temperatures. The bronze corydoras is a social catfish from tropical South American freshwater ecosystems that are prone to seasonal hypoxia (Graham, 1997). It uses its gut as an air-breathing organ and is a ‘non-obligate’ air breather, meaning that, in well-aerated water it does not require air breathing to survive, but it does adopt the strategy under environmental stress (Kramer and McClure, 1980). The bronze corydoras has been reported to perform social air-breathing (Graham, 1997; Kramer and McClure, 1980), although it is not known how environmental conditions may affect this behaviour. We estimated SMR of individuals at two temperatures within the natural range of the bronze corydoras: 25 and 30°C. We then combined this with observations of air-breathing behaviour of individuals and groups in normoxia (100% air saturation), mild hypoxia (60% air saturation) and severe hypoxia (20% air saturation). We aimed to address three main questions: (1) do hypoxia and temperature exert independent and interactive effects on the air-breathing behaviour of individuals in social groups?, (2) do these stressors affect the occurrence of synchronised air breathing? and (3) does an individual's oxygen demand relate to its air-breathing frequency while in a social group?

## MATERIALS AND METHODS

### Study species

Adult bronze corydoras catfish, *Corydoras aeneus* (T. N. Gill 1858), of unknown sex were acquired from an ornamental fish supplier. The fish were semi-domesticated and bred in ponds from wild broodstock. After transport to aquarium facilities at the University of Glasgow (Glasgow, UK), 42 fish (average wet mass 1.651 g, average total length 43.2 mm) were randomly split among six well-aerated tanks (60 cm×40 cm×30 cm) so that each tank held a group of seven individuals. Tanks were supplied with recirculating, UV-treated freshwater at 25°C under a 12 h light:12 h dark photoperiod, and equipped with shelter, plastic plants and a sandy substrate. All neighbouring tanks were divided by an opaque barrier to prevent fish from observing or interacting with individuals from other groups. All fish were acclimated to these conditions for 2 months prior to experiments. At the start of the acclimation period, individuals were sedated using benzocaine and tagged with a unique combination of coloured Visible Implant Elastomer (VIE) tags (Northwest Marine Technology Inc., Shaw Island, WA, USA) for identification during metabolic and behavioural analysis. Fish were fed twice a day with commercial feed and bloodworm and they were fasted 24 h before experiments. The experiments were carried out with the approval of the Home Office under Project Licence no. PB948DAA0.

### Protocol overview

The overall protocol consisted of two main stages. First, after acclimation to laboratory conditions, SMR of each fish (*N*=42) was estimated at 25°C. Then, approximately 1 week later, the behaviour of fish was observed at three oxygen saturations (100%, 60% and 20%), also at 25°C. Second, once all six groups had been tested for metabolic traits and behaviour, the temperature in their holding tanks was increased over a 2 week period to 30°C. After the 2 week period, all fish were tested for SMR and behaviour in the same way as at 25°C.

### Estimates of metabolic rate

The oxygen uptake of each fish was measured using intermittent-flow respirometry to estimate SMR before each round of behavioural observations (Chabot et al., 2016; Svendsen et al., 2016). Individual fish were removed from their holding tanks using a dip net and transferred to individual glass cylindrical respirometry chambers (75 ml volume) attached to an intermittent-flow respirometry system. Seven respirometry chambers were submerged in well-aerated water (4760 cm^2^ surface area, 12.5 cm water depth). Oxygen content of water within the system was recorded every 2 s using a FireSting 4-channel oxygen meter and associated sensors (PyroScience, Aachen, Germany). Water was mixed throughout the respirometry system using a peristaltic pump attached to a timer. Respirometry chambers were flushed with oxygenated water for 2 min every 8 min, and then switched off, sealing the respirometers to measure the rate of oxygen uptake of each fish (Hollins et al., 2019; Thambithurai et al., 2018). Fish were kept in their chambers overnight for a total of 16 h to allow for estimation of SMR. The respirometry set-up was screened behind an opaque sheet to minimise disturbance caused by human presence (Killen et al., 2018; McLean et al., 2018) and a remote desktop was established on a mobile device (RealVNC, Cambridge, UK) to observe oxygen uptake rates remotely, minimising disturbance. Individuals were removed from their chambers the following morning, and were measured for mass (g), total length (mm) and body length (mm). To minimise any oxygen consumption caused via bacterial respiration, the system was bleached daily between trials. Blank measurements using empty chambers were conducted before and after measurements with the fish, and background bacterial oxygen uptake was calculated assuming a linear increase over time and subtracted from the oxygen uptake measures of the individual fish within the respective respirometer. On average, background bacterial oxygen uptake was 16.60±1.68% of that of the fish in the same chamber, across trials. Raw values for SMR were then calculated using LabChart (LabChart v.7.3.8, AD Instruments, Oxford, UK), by determining the slope of oxygen decrease per unit time for each closed phase (omitting the first minute after closure of the respirometer during each cycle). Each slope was then converted to a rate of oxygen uptake after accounting for the total volume of the chamber and associated tubing, after subtracting the volume of the fish. Whole animal SMR (mg O_2_ h^−1^) was estimated as the lowest 20th percentile of measurements taken throughout the trial.

### Behavioural observations

All fish were monitored for air breathing within groups, not in isolation. One week after respirometry, groups were selected for behavioural study using a random number generator. Each group consisted of the seven individuals from a tank, with six groups in total, providing data on individual uptake and behaviour for 42 fish overall. Group observations were performed in a behavioural arena consisting of a circular tank (3117 cm^2^) placed within an outer water reservoir (4760 cm^2^). Water was mixed and aerated in the reservoir, to minimise disturbance to the fish and improve video quality in the arena. The walls of the arena were pierced throughout to allow free exchange of water with the outer reservoir. Oxygen levels were monitored using a galvanic oxygen electrode (OxyGuard Mini Probe, OxyGuard International, Farum, Denmark) placed within the arena. The arena was lined with a white sand substrate to replicate conditions within holding tanks and to stimulate activity of the fish (*Corydoras* species spend much of their time foraging in the substrate). Three small stones were placed in the centre of the arena to act as visual reference points and the set-up was illuminated uniformly with two lamps placed on opposite sides, to enhance visibility. Behavioural observations were recorded using a remotely operated Panasonic 4K video camcorder (Panasonic HC-VX980EB-K) mounted above the arena. The recordings were taken from above as bronze corydoras are bottom dwelling and mainly shoal in two dimensions, so social behaviour can be accurately measured from this angle (Riley et al., 2019).

Groups were placed in the arena in the evening (17:00 h) and allowed to settle and acclimatise to the novel environment overnight. Throughout the night the arena was aerated, and water was normoxic (100% air saturation). The following morning, the lamps were turned on (08:30 h) and the group settled for 30 min. Then, the groups were exposed to progressive hypoxia, followed by recovery to normoxia. Hypoxia levels were controlled using an oxygen regulator (OXY-REG, Loligo, Denmark) attached to a solenoid system. Oxygen content of water was lowered by bubbling 100% nitrogen into the behavioural arena at a rate of 20% every hour. During this time, groups were filmed for 12 min at three oxygen concentration levels: 100%, 60% and 20%. At each step, oxygen content was regulated such that nitrogen was automatically injected into the system with the solenoid whenever water oxygen concentration increased 0.5% above the target oxygen concentration. Fish were given an hour to acclimate to normoxia at the end of each trial before returning to their holding tanks.

### Video analysis

The air-breathing behaviour of fish was observed at each oxygen level, at 25 and 30°C. This resulted in a total of 450 min of footage for six groups and 42 individuals overall. The total number of air breaths performed by a group and the time the air-breathing events occurred were recorded using Solomon Coder (v.17.03.22; Budapest, Hungary), which allows a user to tally time-stamped behavioural events or states from recorded video. Here, an air breath is defined as when a fish darted upwards, broke the surface of the water and ingested air. Within each group, individuals were identified from their unique VIE tags. The number of air breaths taken by each fish was recorded, again using Solomon Coder. In addition, idTracker 2.1 (Pérez-Escudero et al., 2014) was used to obtain spatial trajectories of groups to calculate average speed (cm s^−1^) as a proxy for activity. The cohesion of each group was calculated for every trial as the average distance between each individual; smaller values indicated that individuals were closer to each other and were more cohesive.

To investigate whether air-breathing events were temporally clustered (i.e. the degree of synchronisation), a coefficient of dispersion (CD) was calculated for each 12 min trial (Chapman and Chapman, 1994; Hollins et al., 2019; Killen et al., 2018). Each trial was split into 5 s intervals to reduce the possibility of the same fish being counted twice in a ‘chain’ of clustered breathing events (Hollins et al., 2019; Killen et al., 2018). The mean number of air breaths within each time interval was recorded, along with the variance of each mean. CD was then calculated as the variance/mean ratio across intervals, with values greater than 1 indicating temporally clustered events, and values less than 1 indicating more uniform distributions.

### Statistical analysis

All data are available from Mendeley Data (https://data.mendeley.com/datasets/g68pknt2n9/1). All analyses were conducted in R version 3.5.3 (http://www.R-project.org/) using the lme4 (http://CRAN.R-project.org/package=lme4) and MuMIn (http://cran.r-project.org/package=MuMIn) packages. The effect of acclimation temperature on individual SMR was assessed using a linear mixed-effects model (LME) with log SMR as the response variable, log mass and temperature (categorical) as fixed effects. Individual ID was nested within group and treated as a random factor to account for repeated measures of individuals between temperatures. The effect of environment on group air-breathing behaviour was assessed using a generalised linear mixed-effects model (GLMM) with a negative binomial distribution to account for over-dispersion. The total number of breaths taken by a group was treated as the response variable, with acclimation temperature (25 and 30°C), ambient oxygen level (100%, 60% and 20%), activity and cohesion as fixed factors, and group as a random factor. In addition, the effect of environment on the degree of synchronisation of breathing was assessed using LME. The coefficient of dispersion was treated as the response variable with temperature, ambient oxygen level, activity, cohesion and group number treated as above. Individual variation in air breathing within groups was further analysed using a GLMM with breaths taken by each individual as the response variable, log mass, log SMR, oxygen availability (categorical) and acclimation temperature (categorical) as explanatory variables along with all possible interactions. Fish ID nested within group was included as a random effect in the model. Additional models examined the effect of oxygen availability and acclimation temperature on the activity and cohesion of groups. First, the effect on activity was investigated using LME with speed as the response variable, and cohesion, acclimation temperature and oxygen availability as explanatory variables, and group as a random effect. Second, the effect on cohesion was investigated using LME as above, with cohesion as the response variable.

Models of best fit were determined using likelihood ratio tests (LRT), although if SMR remained in the model, mass was also retained to account for allometric scaling of metabolic rates (Hollins et al., 2019). Assumptions of normality, linearity and homoscedasticity were verified by visual inspection of residual plots. The coefficient of determination, or *R*^2^, was calculated to examine the predictive capacity of models. *R*^2^ values included conditional and marginal *r*^2^ values (*r*^2^_c_ and *r*^2^_m_), which described the proportion of variance explained by fixed factors, and by both fixed and random factors, respectively (Nakagawa and Schielzeth, 2013).

## RESULTS

### Estimates of metabolic rate

**Fig. 1.**
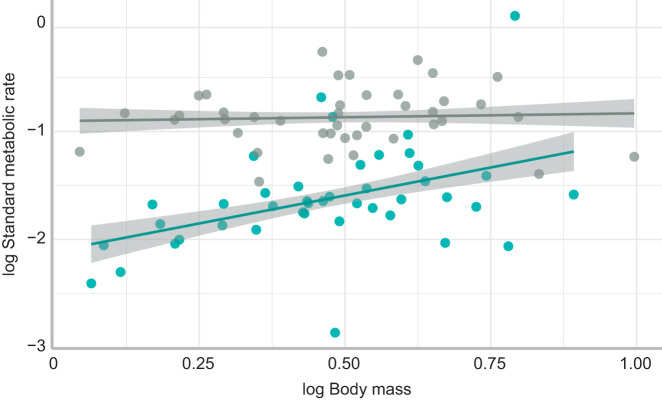
**The relationship between log standard metabolic rate and log body mass in *Corydoras aeneus*.** Each data point represents standard metabolic rate (mg O_2_ h^−1^) against body mass (g) for an individual fish, acclimated at 25°C (blue) and 30°C (grey). The shading around the regression lines represent 95% confidence intervals.

SMR was higher at 30°C compared with 25°C (Table 1; Fig. 1). SMR increased with body mass at 25°C but not at 30°C.

**Table 1.**
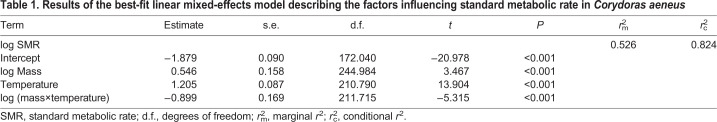
**Results of the best-fit linear mixed-effects model describing the factors influencing standard metabolic rate in *Corydoras aeneus***

### Variation in synchronised air-breathing

**Fig. 2.**
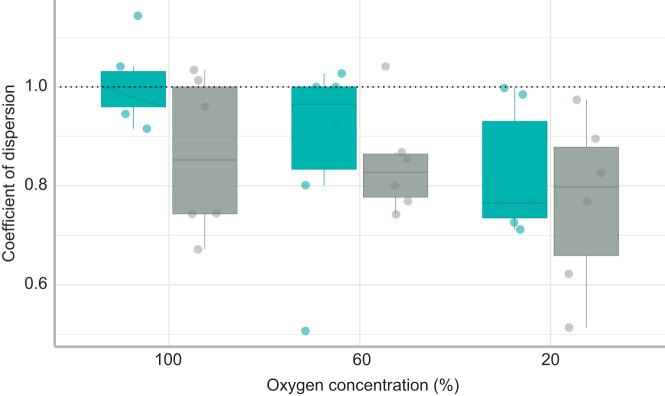
**Clustering of air-breathing events calculated using a coefficient of dispersion at different oxygen concentrations and acclimation temperatures.** Blue bars: 25°C; grey bars: 30°C. Each data point represents the coefficient of dispersion for a trial where any value above 1 indicates that air-breathing events were temporally clustered. The threshold for clustering is shown by the dotted line; data above that line are characteristic of synchronous air-breathing.

Among the 36 trials of group air-breathing behaviour, only six indicated that air-breathing events were temporally clustered (Fig. 2). Of these, half of the clustered trials were within the same group. Overall, occurrences of synchronised air-breathing events occurred across environmental contexts, regardless of acclimation temperature or oxygen availability. However, there was an overall decrease of CD when oxygen levels were decreased during a trial (Fig. 2; Table 2; LME: *t*=2.580, *P*= 0.015).

**Table 2.**
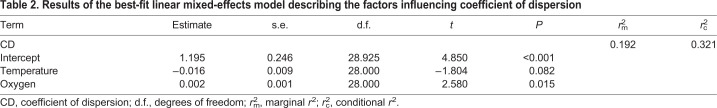
**Results of the best-fit linear mixed-effects model describing the factors influencing coefficient of dispersion**

### Variation in air-breathing frequency

**Fig. 3.**
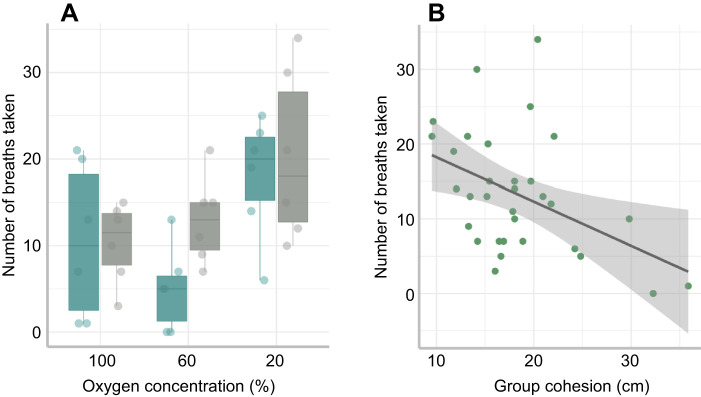
**Factors affecting air-breathing frequency in groups.** (A) Effect of oxygen concentration on air-breathing frequency (ABF) at 25°C (blue) and 30°C (grey). Each data point represents the total ABF for one group of seven fish (*N*=6 groups). (B) Effect of cohesion on ABF; ‘cohesion’ refers to how close individuals were to each other within trials, with lower values indicating more cohesion. Each data point represents the total number of air breaths made by a group, and the shading around the regression line represents a 95% confidence interval. The horizontal line within each boxplot represents the median, the upper and lower limits of the boxplot represent the 75th and 25th percentiles, respectively, and the length of the whiskers represents the range of the data.

Overall, the total number of air breaths taken by each group increased at 30°C (Fig. 3A; Table 3; LME: *z*=2.190, *P*=0.029), and generally increased with progressive hypoxia (LME: *z*=−3.770, *P*<0.001). Regardless of temperature, the total number of air breaths taken by groups was greater at 20% compared with 60% and 100% (46% and 45% more breaths, respectively) (Fig. 3A). The total number of air breaths taken by a group was not affected by group swimming activity (LME: *z*=0.069, *P*=0.945), but the frequency of air breathing increased at the group level when groups were more cohesive (Fig. 3B; LME: *z*=−3.469, *P*=<0.001).

**Table 3.**
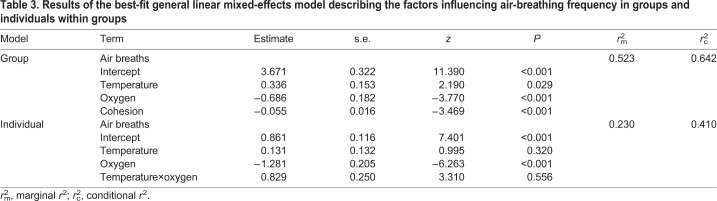
**Results of the best-fit general linear mixed-effects model describing the factors influencing air-breathing frequency in groups and individuals within groups**

**Fig. 4.**
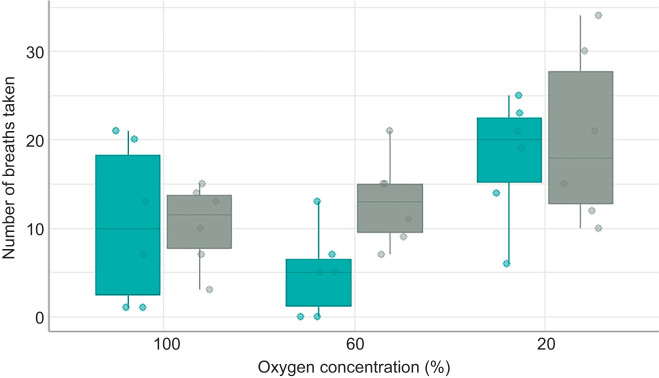
**The effect of oxygen concentration on air-breathing frequency for individual fish at 25 and 30°C.** Blue bars: 25°C; grey bars: 30°C. Each data point represents the total air-breathing frequency for one fish (*N*=42). The horizontal line within each boxplot represents the median, the upper and lower limits of the boxplot represent the 75th and 25th percentiles, respectively, and the length of the whiskers represents the range of the data.

Air breaths were performed at all oxygen availabilities but air-breathing frequency (ABF) for individuals was greatest at 20% oxygen availability (Fig. 4; Table 3; LME: *z*=−6.263, *P*<0.001). There was no effect of temperature on an individual's ABF. Although individuals acclimated to 30°C took more air breaths at 60% oxygen availability compared with at 25°C, this difference was not statistically significant (Table 3; LME: *z*=3.310, *P*=0.556). There was no relationship between an individual's ABF and their SMR (LME: *z*=−0.501, *P*=0.616).

### Variation in group dynamics: activity and cohesion

**Fig. 5.**
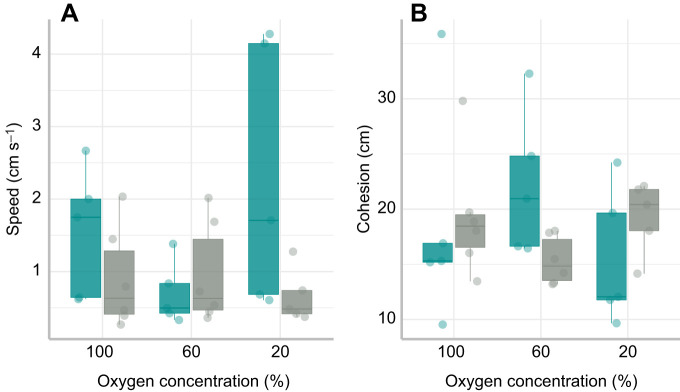
**Factors affecting activity and cohesion in groups.** (A) Effect of oxygen concentration on activity (using speed as a proxy) at 25°C (blue) and 30°C (grey). Each data point represents the total speed for one group of seven fish (*N*=6 groups). (B) Effect of oxygen concentration on cohesion at 25°C (blue) and 30°C (grey). Each data point represents the cohesion for one group of seven fish (*N*=6 groups), where smaller cohesion values indicate that individuals within a group were closer together, thus were more cohesive. The horizontal line within each boxplot represents the median, the upper and lower limits of the boxplot represent the 75th and 25th percentiles, respectively, and the length of the whiskers represents the range of the data.

Additional models examined the effect of acclimation temperature and oxygen availability on group activity and cohesion (Table 4). The average speed of groups was greatest at 25°C compared with 30°C when the availability of oxygen was at 100% and 20% (Fig. 5A). However, the average speed of groups was significantly less at 60% oxygen availability (Fig. 5A). Thus, there was a significant interaction between temperature and oxygen availability (Table 4; LME: *t*_25_=2.335, *P*=0.028).

**Table 4.**
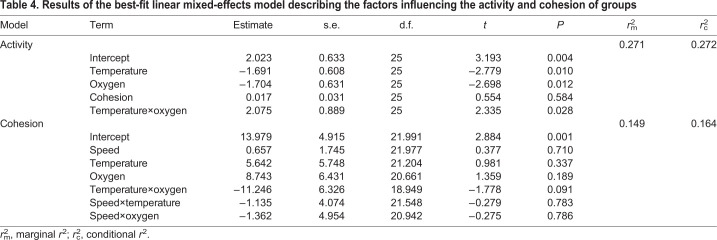
**Results of the best-fit linear mixed-effects model describing the factors influencing the activity and cohesion of groups**

The cohesion of groups was not affected by temperature (LME: *t*_25_=0.981, *P*=0.337) or oxygen (LME: *t*_25_=1.359, *P*=0.189). There was weak evidence for an interaction between temperature and oxygen at 60% oxygen availability, although this was not statistically significant (Fig. 5B; LME: *t*_25_=−1.778, *P*=0.091). There was also no relationship between speed and cohesion at either temperature (Table 4).

## DISCUSSION

Contrary to previous reports, the results presented here provide limited evidence that bronze corydoras catfish perform synchronised air breathing under any environmental condition examined. Interestingly, however, groups that were more cohesive while performing routine activities tended to take more air breaths overall. While oxygen availability affected the air-breathing frequency of both groups and individuals, the impact of acclimation temperature was less conclusive. Although acclimation temperature resulted in an increase in SMR, the oxygen demand of an individual had no effect on its air-breathing frequency in a group. These observations highlight that while oxygen availability is a clear driver of air breathing, the behaviour of groups, and specifically social cohesion, can also have important consequences for air-breathing behaviour. Thus, these results provide an example of how social effects on individual behaviour can seemingly supersede individual physiological demands when acquiring a resource.

### Synchronised air breathing

There was an overall trend of decreasing CD as oxygen availability decreased, which may be indicative of increased temporal homogeneity between breaths among individuals as the trial progressed. Nevertheless, there was limited evidence for clustering of air-breathing behaviour. Only six trials indicated that air breathing was synchronous among individuals within a group. This contrasts with the findings of Kramer and McClure (1980) who first described social air-breathing in the bronze corydoras. In the current study, air breaths were described as synchronous if individuals breathed air simultaneously or within a specific time frame. However, in Kramer and McClure (1980), if conspecifics accompanied an individual to the surface, but only one of them breathed air, this would still be considered synchronous air-breathing. Thus, it is possible that earlier studies may have over-estimated the occurrence of synchronous air-breathing in this species. Similarly, early studies employed a more qualitative approach when describing synchronous air-breathing. If individuals performed air breaths simultaneously at some points in the trial, they were described as synchronous air breathers (Graham, 1997; Kramer and Graham, 1976). However, within a trial, individuals can also perform air breaths alone. If over the course of the trial individuals perform air breaths on their own, more often than they do in groups, the distribution of air-breathing events throughout is more temporally uniform.

An alternative explanation for the lack of synchronous air-breathing in the current study is that synchronous air-breathing may provide an anti-predator function and individuals may be more likely to breathe air together if they perceive there is a risk of predation (Chapman and Chapman, 1994; Kramer and Graham, 1976). Thus, the lack of synchronous air-breathing may be indicative of a lack of perceived threat. While there was no risk of predation in the current study, other studies have found synchronous air-breathing in the absence of predation. Killen et al. (2018) observed synchronous air-breathing in a behavioural arena when decreasing the amount of oxygen available. Although the set-up was similar to the one used in the current study, Killen et al. (2018) studied the African sharptooth catfish *Clarias gariepinus*. The difference in synchronous air-breathing reported by the studies may thus be due to interspecific anti-predator strategies. For example, some other air-breathing fishes have developed alternative predator avoidance behaviours such as minimising time at the surface (Kramer and Graham, 1976). More study is needed, therefore, to determine whether the bronze corydoras uses synchronous air-breathing to avoid predation, or if they use an alternative strategy. It is also worth noting that the fish used in the current study are unlikely to have experienced predator exposure, having been bred in captivity. It is therefore possible that this may have affected their risk perception in the experimental arena.

### Variation in air-breathing frequency

Fish breathed air at all oxygen availabilities, but frequency was highest at the most extreme level of hypoxia. This is consistent with other studies that have examined the impact of oxygen availability (da Cruz et al., 2013; McKenzie et al., 2016; Randle and Chapman, 2005; Scott et al., 2017). There was also an impact of temperature on ABF, although this was less marked. Overall, groups had a higher ABF after the 2 week acclimation to 30°C, especially at 20% oxygen availability. This may be due to hypoxia being exacerbated at higher temperatures due to a reduction in dissolved oxygen or elevated metabolic rate, which may have increased oxygen demand (Breitburg et al., 2018; da Silva et al., 2017; Graham, 1997). Wang and Lin (2018) compared ABF under hypoxia alone, temperature alone, and then both stressors combined and found that ABF was highest at the most extreme temperature and hypoxia combination. However, Wang and Lin (2018) only found an effect after exposing fish to each treatment for a minimum of 2 h. Therefore, future studies could determine whether exposure time affects the ABF of the bronze corydoras.

It is interesting that the ABF of groups was also affected by social cohesion, in that the more cohesive the group the greater the ABF. In bronze corydoras, individuals that are close to each other can better utilise social cues and may also perceive less risk because there are other individuals in their immediate vicinity (Riley et al., 2019). A more remote possibility could be that, if individuals are closer together, they may reduce the amount of oxygen available for others in the group (Nadler et al., 2016). Thus, air breathing could be a potential adaptive strategy in populations that are closely aggregated. The trade-off between air breathing and population density could therefore be an interesting future direction of study.

Although there were group-level differences in ABF in response to temperature, there was no effect of temperature on air breathing at the individual level. This may be due to variation among groups, with temperature having a disproportionate effect on some groups compared with others. There was also no relationship between an individual's ABF within groups and their individual oxygen demand. Although acclimation to 30°C resulted in a higher SMR, this had no effect on an individual's tendency to breathe air. This contradicts previous studies, which found a relationship between oxygen demand and air breathing during normoxia, and especially so in hypoxia (McKenzie et al., 2016). However, Killen et al. (2018) also found no association between SMR or tendency to breathe air among individuals. Instead, they found that behaviour, namely aggression, was the main factor influencing air breathing. Although there was no impact of activity on air-breathing behaviour in this study, and no notable instances of aggression, this is probably due to differences in the ecology of the study species used. *Clarias gariepinus*, used by Killen et al. (2018), is an aggressive catfish, which forms social hierarchies in the wild that are driven by dominance. It is not known whether bronze corydoras form social hierarchies in the wild or if they display differences in personality traits such as dominance, but a social network approach could help us understand whether these traits can modulate air-breathing behaviour in this species. In addition, social stimuli may affect individual oxygen demand via effects on endocrine pathways or stress responses, in a manner that over-rides any influence of SMR on air breathing among individual fish (Culbert et al., 2019; Nadler et al., 2016). It is also possible that exposure to hypoxia during the trials at 25°C may have affected individual air-breathing tendency when later tested at 30°C.

### Conclusion

The current data contribute to our understanding of how environmental stressors may have an impact on behaviour. However, the findings indicate that the effects of environmental stressors on air-breathing behaviour are complex. In this study, *C. aeneus* were not always synchronous in their air-breathing behaviour. It is possible that some species may perform synchronised air breaths less than was initially reported, which may at least in part reflect how the phenomena are evaluated. The study also demonstrates that multiple stressors can affect air-breathing behaviour, with air-breathing frequency increasing at the most extreme temperature and hypoxia combination. The impact of this, however, differs between groups and individuals. Therefore, it is important to consider how climate-related stressors may have an impact on air-breathing fishes at multiple levels. Finally, this study demonstrates that individual physiology may not explain variation in an individual's air-breathing frequency. Thus, under environmental stress, other factors may over-ride the potential effects of physiology on behaviour. Social cohesion, for example, is a clear example of the importance of group behaviour in an ecological context, and it provides an exciting future direction for air-breathing research even if social cohesion during routine activities does not always translate into synchronised air-breathing *per se*. Overall, the results from this study indicate that there are context-dependent complexities at play that can influence how the environment may affect risky behaviour when acquiring a resource.
